# Effectiveness of Artificial Intelligence Models in Predicting Lung Cancer Recurrence: A Gene Biomarker-Driven Review

**DOI:** 10.3390/cancers17111892

**Published:** 2025-06-05

**Authors:** Niloufar Pourakbar, Alireza Motamedi, Mahta Pashapour, Mohammad Emad Sharifi, Seyedemad Seyedgholami Sharabiani, Asra Fazlollahi, Hamid Abdollahi, Arman Rahmim, Sahar Rezaei

**Affiliations:** 1 Student Research Committee, Tabriz University of Medical Sciences, Tabriz 5165665931, Iran; pourakbarn@tbzmed.ac.ir (N.P.); a.motamedi@tbzmed.ac.ir (A.M.); pashapourm@tbzmed.ac.ir (M.P.); gholamisharabiani@tbzmed.ac.ir (S.S.S.); fazlollahia@tbzmed.ac.ir (A.F.); 2 Shariati Hospital Research Center, Tehran University of Medical Sciences, Tehran 1416634793, Iran; me-sharifi@student.tums.ac.ir; 3 Research Department of Integrative Oncology, BC Cancer Institute, Vancouver, BC V5Z 1L3, Canada; habdollahi@bccrc.ca (H.A.); arman.rahmim@ubc.ca (A.R.); 4 Departments of Radiology and Physics, University of British Columbia, Vancouver, BC V5Z 1M9, Canada; 5 Department of Radiology, Medical School, Tabriz University of Medical Sciences, Tabriz 5165665931, Iran

**Keywords:** lung cancer recurrence, gene biomarkers, artificial intelligence, predictive models, clinical integration

## Abstract

Background/Objectives: Lung cancer recurrence, particularly in NSCLC, remains a major challenge, with 30–70% of patients relapsing post-treatment. Traditional predictors like TNM staging and histopathology fail to account for tumor heterogeneity and immune dynamics. This review evaluates AI models integrating gene biomarkers (TP53, KRAS, FOXP3, PD-L1, and CD8) to enhance the recurrence prediction and improve the personalized risk stratification. Methods: Following the PRISMA guidelines, we systematically reviewed AI-driven recurrence prediction models for lung cancer, focusing on genomic biomarkers. Studies were selected based on predefined criteria, emphasizing AI/ML approaches integrating gene expression, radiomics, and clinical data. Data extraction covered the study design, AI algorithms (e.g., neural networks, SVM, and gradient boosting), performance metrics (AUC and sensitivity), and clinical applicability. Two reviewers independently screened and assessed studies to ensure accuracy and minimize bias. Results: A literature analysis of 18 studies (2019–2024) from 14 countries, covering 4861 NSCLC and small cell lung cancer patients, showed that AI models outperformed conventional methods. AI achieved AUCs of 0.73–0.92 compared to 0.61 for TNM staging. Multi-modal approaches integrating gene expression (PDIA3 and MYH11), radiomics, and clinical data improved accuracy, with SVM-based models reaching a 92% AUC. Key predictors included immune-related signatures (e.g., tumor-infiltrating NK cells and PD-L1 expression) and pathway alterations (NF-κB and JAK-STAT). However, small cohorts (41–1348 patients), data heterogeneity, and limited external validation remained challenges. Conclusions: AI-driven models hold potential for recurrence prediction and guiding adjuvant therapies in high-risk NSCLC patients. Expanding multi-institutional datasets, standardizing validation, and improving clinical integration are crucial for real-world adoption. Optimizing biomarker panels and using AI trustworthily and ethically could enhance precision oncology, enabling early, tailored interventions to reduce mortality.

## Simple Summary

Lung cancer, one of the most prevalent cancers worldwide, representing about 11.6% of all newly diagnosed cancer cases, is the leading cause of cancer-related deaths. Recurrence of lung cancer occurs in a significant proportion of patients, particularly in patients with non-small cell lung cancer, with rates ranging from 30% to 70% after initial treatment. This study aims to assess artificial intelligence models predicting lung cancer recurrence by integrating genomic biomarkers, thereby improving the personalized risk evaluation.

## 1. Introduction

Lung cancer burdens global health, particularly in men, with high rates of occurrence and death [1,2]. It continues to be the primary cancer-driven cause of death in many areas, even with declining mortality rates [1]. Tobacco smoking is the top risk factor. Studies show that following steps to quit smoking can reduce the lung cancer risk by 87% in non-smokers and 45% in light smokers compared to heavy smokers [3]. Along with smoking, following cancer prevention tips from the World Cancer Research Fund/American Institute Cancer Research (WCR/AICR) can help reduce the risk [3]. Early cancer detection through standard checks helps improve survival rates by aiding in the early detection of localized disease [1]. Yet, lung cancer often shows up late clinically, resulting in a low survival rate over five years, most notably with non-small cell lung cancer (NSCLC) [2]. While significant advancements have taken place in diagnostics and treatments such as surgery, chemotherapy, specific therapies, and radiotherapy [2,4], ensuring equal access to these breakthroughs remains a pervasive issue.

Estimating lung cancer recurrence relies on clinical, pathological, molecular, and imaging-based strategies. Clinical assessments include the TNM staging system, which evaluates the tumor size, lymph node spread, and distant metastasis to gauge the recurrence risk [5], alongside tumor histology and grade, where cellular abnormalities and growth patterns inform the prognosis [6]. Surgical outcomes, such as margin clearance after tumor removal, also correlate with the recurrence likelihood [5]. Molecular methods analyze genetic and protein activity, with gene expression profiling identifying metastasis-linked genes [7] and protein biomarkers like those regulating cell death offering predictive insights [7]. Immunohistochemistry further detects proteins such as FOXP3 in tumor tissues, which may signal a higher recurrence potential [8]. Imaging techniques, such as a CT-based radiomic analysis, evaluate tumor characteristics like opacity, shape, textures, and density, integrating clinical data to enhance the outcome prediction [6,9]. While these methods offer valuable insights, their accuracy often falls short compared to AI-driven models, which integrate diverse data types for improved precision. Based on some evidence, even though a radiomic analysis extracts valuable features from medical images, it often fails to fully capture tumor heterogeneity or provide detailed molecular insights, limiting its predictive accuracy [10]. Gene expression profiling, while insightful, requires invasive tissue sampling, making it costly and impractical for all patients [11]. Similarly, relying solely on clinical and pathological factors may miss critical molecular or genetic predispositions to recurrence [12], as these methods do not always account for the complex biological mechanisms underlying cancer progression. These conventional methods prioritize static anatomical or cellular features (e.g., tumor size and lymph node involvement) but fail to account for dynamic biological processes, such as immune evasion or epigenetic reprogramming, which drive cancer progression and relapse.

Certain genetic markers like *IGFR1* expression, metalloproteinases (MMPs), and changes in the *APC*, *TP53*, and *KRAS* genes might indicate if cancer will return [13]. IGFR1 seems to predict the recurrence of lung cancers called adenocarcinomas after surgery [13], and MMPs appear to show if the other type of lung cancer (non-small cell) will come back after surgery [14]. Changes in the APC, TP53, and KRAS genes often show up in lung cancer patients. Changes in the TP53 gene are usually seen in patients with lung metastasis and a large number of small tumors [15]. MicroRNAs might be used for diagnosis, but how to use them in a clinic needs to be standardized [16]. New methods like overall genetic profiling using next-generation sequencing (NGS) [15] and tracking immune checkpoint blockade (ICB) markers [17] might give us a clearer picture of someone’s genes and help make treatment decisions.

AI is a multidisciplinary field focused on automating tasks that typically require human intelligence, such as decision-making, problem-solving, and language processing. It leverages large datasets and sophisticated algorithms to perform these tasks efficiently and optimally, often surpassing human capabilities in speed and accuracy [18].

AI models, such as artificial neural networks (ANNs), have demonstrated robust performance in forecasting lung cancer recurrence by analyzing complex datasets [19]. Complementing these approaches, quantitative imaging techniques—including CT and PET-CT scans—enable clinicians to measure tumor growth kinetics, such as metabolic activity and volumetric doubling times, which correlate strongly with the relapse risk [20]. Beyond structural and metabolic insights, AI tools further enhance the predictive accuracy by integrating gene biomarkers, such as differentially expressed immune-related genes (e.g., RLTPR and SLFN13), which are imperceptible to conventional imaging or histopathology [21]. For instance, multilayer perceptron networks have achieved >89% accuracy in validation cohorts by synthesizing genomic, radiomic, and clinical data, underscoring AI’s capacity to decode multifaceted biological drivers of recurrence [19].

Researchers have leveraged AI techniques to identify differentially expressed genes (DEGs) that serve as promising indicators for lung cancer recurrence [21]. For example, one particular investigation found 37 DEGs between primary and recurrent lung adenocarcinoma (LUAD) tumors, with 31 DEGs significantly associated with recurrence-free survival (RFS) [21]. Another study discovered five immune-related genes—RLTPR, SLFN13, MIR4500HG, HYDIN, and TPRG1—that showed a strong relationship with the early recurrence of stage Ia-b NSCLC [22]. Additionally, the CIBERSORT (Cell-type Identification by Estimating Relative Subsets Of RNA Transcripts) algorithm measures tumor-infiltrating immune cells (TIICs). It pinpoints activated natural killer (NK) cells, M0 macrophages, M1 macrophages, and T CD4+ memory resting cells as possible recurrence indicators in patients with early-stage lung cancer [23].

AI methodologies have been instrumental in developing predictive frameworks for lung cancer to forge a cutting-edge prognostic tool. Researchers have strategically combined LASSO Cox regression with multivariate Cox proportional hazards modeling. This integrative approach pinpointed a concise 13-gene signature capable of forecasting recurrence-free survival in LUAD patients. The model’s robustness was recurrence using gene expression data and clinical traits; for instance, a study employing a multilayer perceptron neural network flaunted an impressive predictive accuracy of 87.5%, 89.1%, and 89.9% for the training, validation, and test datasets, respectively [19]. It was rigorously vetted through iterative internal validation and replicated across independent external cohorts, demonstrating remarkable generalizability in diverse clinical settings [21]. Another researcher crafted an ensemble linear kernel support vector machine (SVM) ML model to forecast tumor recurrence of early-stage lung cancer using optimized clinical and genomic attributes, achieving exceptional precision [23]. Yet, despite these positive outcomes, numerous hurdles still lurk ahead. The costs and invasiveness of gene collection; the necessity for larger, varied databases; and merging gene expression data with other data formats, like radiomic features from CT scans, remain a challenge [11,24,25].

Past strategies for predicting lung cancer recurrence, such as TNM staging or histopathological assessments, focused heavily on fixed clinical features like the tumor size or cellular structure. These methods, while foundational, often missed the nuanced biological shifts driving cancer progression. Molecular approaches, including gene expression profiling, added deeper insights but faced hurdles like inconsistent results across patient groups and the need for invasive tissue sampling. For example, traditional radiomics could map tumor shapes on scans, but struggled to decode how tumors interact dynamically with their microenvironment. This review shifts the focus by exploring how AI models merge genetic biomarkers—such as IGFR-1 or TP53 mutations—with clinical and imaging data, creating a more holistic view of the recurrence risk. Unlike older methods that treated these datasets in isolation, AI tools like neural networks uncover hidden patterns, such as how immune cells infiltrating tumors (e.g., NK cells or macrophages) influence relapse. By analyzing studies from diverse global settings, we highlight how these models outperform conventional techniques, achieving over 89% accuracy in some cases. Our work also addresses persistent challenges, like small sample sizes and data variability, by proposing collaborative frameworks, such as federated learning, to pool data securely across institutions. This approach not only improves the prediction accuracy but also paves the way for clinically adaptable tools that balance precision with practicality, ultimately helping clinicians tailor follow-up care to individual patient risks.

## 2. Materials and Methods

This review was conducted by following the Preferred Reporting Items for Systematic Reviews and Meta-Analyses (PRISMA) recommendations [26].

### 2.1. Search Strategy

A comprehensive search was conducted to examine how AI tools are utilized to identify genetic markers linked to lung cancer recurrence in September 2024. Databases such as PubMed, Embase, Cochrane Library, Scopus, Web of Science, and Google Scholar were scanned using customized search strategies to capture relevant publications. The search criteria focused on three themes: (1) AI methods (e.g., “machine learning”, “neural networks”, and “deep learning”), (2) lung cancer terminology (e.g., “lung carcinoma” and “pulmonary tumors”), and (3) recurrence indicators (e.g., “relapse” and “recurrent disease”). Each platform’s unique search features were accommodated; for example, PubMed queries integrated Medical Subject Headings and logical operators like “AND” to merge concepts, while Scopus and Web of Science searches filtered results using the title, abstract, and keyword fields. Google Scholar relied on a streamlined approach, blending broad terms like “AI” and “lung cancer recurrence” to identify studies. This approach aimed to minimize gaps in retrieving studies that bridge biomarker discovery, oncology, and AI-driven analytics.

### 2.2. Eligibility Criteria

The primary studies that developed machine learning or deep learning models to predict lung cancer recurrence by genomic biomarkers were included in this review. In vitro models as well as reviews/meta-analyses, editorials, letters, and invited opinions, were excluded. In addition, articles not available in English and those referring to organisms other than humans were excluded.

### 2.3. Study Selection

The study selection process involved two sequential phases. Initially, two trained reviewers (N.P. and M.P.) independently screened the titles and abstracts of the retrieved records via EndNote (version 21.3, 2023) to identify studies meeting the preliminary eligibility requirements. Eligible articles were then subjected to a full-text evaluation by the same reviewers, applying predefined criteria: inclusion was restricted to peer-reviewed studies in English or Persian that specifically evaluated AI-driven models for predicting lung cancer recurrence using genomic biomarkers (e.g., gene expression profiles or mutations) while excluding non-AI methods, studies focused on non-lung cancers, research relying exclusively on non-genomic data (e.g., imaging), and non-empirical publications (e.g., editorials or abstracts). Articles failing to integrate genomic data into AI frameworks were systematically excluded during the secondary screen. Discrepancies between reviewers were resolved through consensus or input from a third arbitrator, ensuring methodological rigor and alignment with the study’s focus on AI–genomic synergy in recurrence prediction.

### 2.4. Data Extraction

A structured template guided the systematic extraction of information from the selected studies. One researcher entered the data into a spreadsheet, which was then reviewed independently by two other team members to confirm consistency and minimize errors. Key details captured included study authorship, publication year, research design, cancer classification (type and stage), participant demographics (sample size, average age, and gender distribution), and technical aspects of analytical models, such as methods for feature selection, training protocols, and evaluation measures (e.g., AUC/ROC values, sensitivity-specificity ratios, accuracy, and Dice/F1 scores). Additional recorded elements covered data profiles, critical biomarkers, the geographic origin of the study, the sample cohort size, timeline, the machine learning frameworks applied, and population characteristics (age range and sex). This organized process ensured the consistent documentation of both clinical and computational variables for comparative analysis.

### 2.5. Data Synthesis

The extracted data were narratively synthesized and presented in tables and figures. Due to the heterogeneity of the included studies, a meta-analysis was not performed. The results were discussed in the context of the existing literature, and the strengths and limitations of the review were highlighted.

## 3. Results

### 3.1. Demographic Characteristics

**Figure 1:**
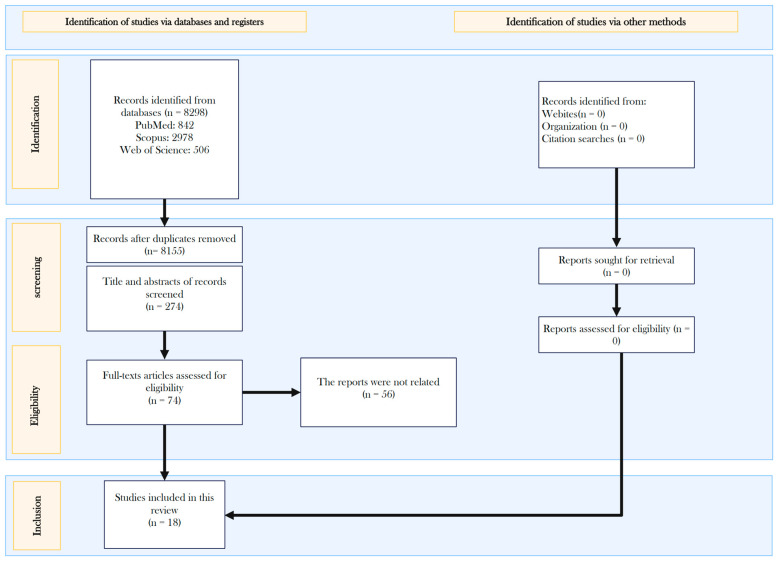
Flowchart of study selection.

A total of 3298 articles were initially identified through database searches using specific keywords. After removing duplicates, 2702 articles remained and were screened based on their titles. From these, 274 studies were selected for the abstract review. The full texts of 74 studies were then independently evaluated for eligibility by two authors. Fifty-six studies were excluded due to their unrelatability, and, ultimately, 18 studies met the criteria and were included in the meta-analysis (Figure 1) [7,8,21,23,25,27,28,29,30,31,32,33,34,35,36,37,38,39].

The included studies consisted of analytical predictive studies, prospective cohort studies, and retrospective observational studies published from 2019 to 2024. In total, the studies included 4861 patients, with sample sizes ranging from 41 to 1348 participants. The mean age span of participants across the studies ranged from 33 to 86 years.

The gender distribution showed that men accounted for a significantly larger proportion of participants (3228) compared to women (1633) in studies that reported these data. The reported median follow-up duration of studies ranged from a minimum of 3 years to a maximum of 10 years.

The review covered multiple lung cancer subtypes (Table 1). NSCLC was the most researched type, featured in seven studies, with the largest single study containing 827 participants. Given its prevalence, NSCLC received the most attention. Among its subtypes, lung adenocarcinoma was specifically investigated in six studies, totaling 3026 patients. Small cell lung cancer (SCLC) was less represented, appearing in just one study with 102 patients.

Studies were conducted in various countries, reflecting the diverse genetic backgrounds among participants. Seven studies were conducted in China, three in the United States, two in UK, and one study each in France, India, Korea, Japan, Sweden, Canada, Iraq, Ireland, Spain, and the Czech Republic.

### 3.2. Machine Learning Techniques

**Figure 2:**
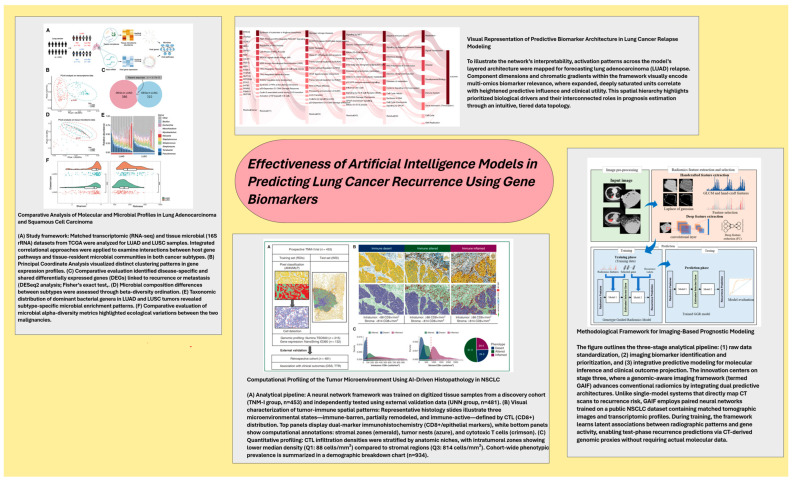
Explanation of curated visuals containing methodological details from reference articles.

A variety of machine learning techniques were evaluated (Table 1), with support vector machine (SVM) and regression models (Lasso and Cox) being the most often utilized, each appearing in four papers. This review also includes single instances of additional well-known techniques such as Random Forest, Gradient Boosting, neural networks, K-Nearest Neighbors, and naive Bayes, displaying a comprehensive exploration of ML methodologies. Interestingly, the studies contained experiments utilizing custom-developed models such as s-DeepBTS, su-DeepBTS, and an Optuna-based XGBoost model. In total, at least 12 unique ML approaches were reported among the investigations (Table 1) (Figure 2).

#### Model Performance

A series of the included studies published in recent years demonstrates the transformative role of machine learning (ML) algorithms in enhancing the recurrence prediction for lung cancer by integrating gene expression profiles with clinical characteristics.

- Gradient Boosting and XGBoost Model

For instance, Jones et al., 2021 [28] developed the PRecur model, a gradient-boosted machine learning framework that combines clinicopathologic variables such as the tumor size and histological subtype with genomic data like TP53 and SMARCA4 mutations. This model achieved a concordance probability estimate (CPE) of 0.73, significantly outperforming the conventional TNM classification system (CPE = 0.61) [28]. Similarly, in the study by Jiang et al., an immune risk score was built based on FOXP3, PD-L1 on TILs, and CD8 markers using the XGBoost algorithm that achieved an AUC of 0.866 and was superior to single-indicator models. When the immune risk score derived from these markers was combined with clinical staging, the predictive accuracy improved further, with AUCs of 0.656, 0.737, and 0.698 for 1-, 3-, and 5-year relapse-free survival (RFS), respectively [8].

- Support Vector Machines and Regression Models

In addition, a support vector machine (SVM)-based combo-classifier that integrated clinical and gene expression data showed exceptional performance, achieving an AUC of 92.0% (95% CI: 89.0–95.0%), which markedly outperformed models relying solely on clinical parameters [23]. 

A multivariable Cox regression analysis identified CDC20 as both an independent prognostic factor and its robust predictive power when combined with traditional clinical characteristics [30]. Additionally, a machine learning-based immunophenotyping model for STK11/KEAP1 co-mutations improved prognostic predictions, yielding AUCs of 0.58 for disease-specific survival (DSS) and 0.56 for the time to recurrence, and further demonstrated additive value to TNM staging [31].

- Hybrid and Feature-Enriched Models

Moreover, integrating imputed aneuploidy scores with clinical features improved the prediction model’s AUC from 0.78 to 0.79 [34], while incorporating imputed pathway scores yielded a further increase to 0.80 using a Random Forest model [35].

The study by Wang et al. introduced an AI-driven bioinformatic and statistical model that identified RBBP7 and YEATS2 as key acetylation-related genes and developed the Acetylation-Related Score (ARS) to predict the recurrence of early-stage LUAD. ARS outperformed clinical features in the recurrence prediction, achieving pooled HR = 1.88 (*p* < 0.001) and AUCs of 0.679, 0.669, and 0.600 for 1-, 3-, and 5-year recurrence-free survival [36].

For squamous cell lung carcinoma (LUSC) patients, AI-driven models also improved the predictive accuracy by combining mRNA and microbiome data, and the tumor stemness and immune infiltration-specific signature (TSISig) with clinical to traditional clinical features [33,38]. Interestingly, the study by Abdu-Aljabar et al., 2023 introduced a hybrid model leveraging XGBoost with Optuna hyperparameter optimization to enhance the prediction of lung cancer recurrence using gene expression datasets. It extracted specific genes (BTBD6, KLHL7, and BMPR1A) as predictive biomarkers and achieved accuracies of 93% and 81% on the respective datasets, outperforming traditional machine learning algorithms such as SVM and naïve Bayes [27].

### 3.3. Deep Learning and Multi-Omics Integration

Most studies utilized machine learning models like gradient boosting, XGBoost, Random Forest, SVM, and Cox regression, enhancing the recurrence prediction by integrating gene features and omics data with clinical variables. Meanwhile, the deep learning models used in three studies advanced multi-omics integration for improved prediction.

For instance, Aonpong et al., 2021 proposed the Genotype-Guided Radiomics framework, which combines radiomics features from CT images with estimated gene expressions. By fusing handcrafted and deep-learning-derived features, the GGR model achieved a recurrence prediction accuracy of 83.28%, which was significantly superior to conventional radiomic methods (78.61%) [25].

Similarly, the BPN-ALO AI model demonstrated a robust capability to integrate dimensionality reduction, feature optimization, and a neural network structure, achieving superior accuracy, sensitivity, and specificity in predicting lung cancer recurrence compared to traditional approaches [32].

Another notable advancement is the IBPGNET framework, a deep learning model that integrates multi-omics data and latent biological pathway relationships for predicting lung adenocarcinoma (LUAD) recurrence. IBPGNET achieved an AUC of 0.88, outperforming algorithms like Random Forest, SVM, PathCNN, and DeepOmix. By combining data from copy number variations (CNVs) and single nucleotide variants (SNVs), it enhanced the predictive accuracy, with the integration of SNV + AMP_CNV + DEL_CNV yielding the highest AUPR of 0.79. Additionally, IBPGNET’s use of graph neural networks and hierarchical visualization identified key genes and pathways, such as PSMC1 and PSMD11, contributing to LUAD recurrence and drug resistance [37].

### 3.4. Key Features and Gene Biomarkers

This systematic review identified several key gene biomarkers that significantly enhance AI-driven models for predicting lung cancer recurrence (Table 2) and summarizes them in Table 3. Various studies have highlighted distinct sets of predictive genes, emphasizing the importance of integrating molecular markers with AI techniques to improve the prognostic accuracy.

#### 3.4.1. Biomarkers for LUAD/NSCLC Prediction

- Zhong et al., 2019 [7] identified PDIA3, MYH11, PDK1, SDC3, RPE65, LAMC3, BTK, and UPK1B as key predictive biomarkers.
- Jones et al., 2021 [28] found that SMARCA4, TP53, and genomic alterations measured by the Fraction of Genome Altered (FGA) were significant recurrence predictors.
- Luo et al., 2020 [29] reported that CpG methylation markers, including ART4, KCNK9, FAM83A, and C6orf10, provided valuable prognostic insights.
- Xu et al., 2020 [21] identified a 12-gene signature (ACTR2, ALDH2, FBP1, HIRA, ITGB2, MLF1, P4HA1, S100A10, S100B, SARS, SCGB1A1, SERPIND1, and VSIG4) that demonstrated strong predictive capability.

#### 3.4.2. Immune-Related Markers

- Jiang et al., 2021 [8] found that FOXP3 expression and PD-L1 on tumor-infiltrating lymphocytes (TILs) played crucial roles in predicting SCLC recurrence.
- Rakaee et al., 2023 [31] reported that STK11 and KEAP1 co-mutations were associated with distinct immune phenotypes, impacting the recurrence risk.

#### 3.4.3. Multi-Omics Approaches

- Xu et al., 2024 [37] demonstrated that the integration of PSMC1, PSMD11, PRKCB, CCNE1, NRG1, ZNF521, and NGF significantly improved the predictive accuracy.
- Zhou et al., 2023 [38] identified the long non-coding RNAs (lncRNAs) LINC00675 and MEG3 as critical recurrence predictors.
- Shi et al., 2021 [33] reported CPS1, CCR2, NT5E, ANLN, and ABCC2 as biomarkers with strong prognostic value.

#### 3.4.4. Tumor and Immune Markers

- Shen et al., 2023 [23] identified MR1, BCL6, and CCL13 in tumor tissues and TBX21, IL-17RB, and GZMB in the buffy coat as key recurrence predictors.
- Abdu-Aljabar et al., 2023 [27] found that BTBD6, KLHL7, and BMPR1A were highly predictive of lung cancer recurrence.

#### 3.4.5. Integration with AI for Enhanced Prediction

The combination of these biomarkers with clinical data and machine learning approaches has significantly improved the predictive accuracy. Across various studies, AI-driven models incorporating these molecular features achieved AUC values ranging from 0.76 to 0.965. These findings underscore the transformative potential of integrating advanced AI techniques with genomic and immune markers to enhance the lung cancer recurrence prediction, paving the way for more personalized and precise risk stratification.

### 3.5. Validation and Generalizability

The studies reviewed in this work employed diverse machine learning algorithms and biomarkers for predicting lung cancer recurrence, with varying performance metrics across AUC/ROC, sensitivity, and specificity. Notably, Zhong et al., 2019 [7] developed a support vector machine model using recursive feature elimination, achieving an AUC of 0.95 with a sensitivity of 0.88 and specificity of 0.90. The model identified key biomarkers, including PDIA3, MYH11, and PDK1.

Abdul-Aljabar et al., 2023 [27] implemented an Optuna-optimized XGBoost model that demonstrated strong performance, with a sensitivity of 1.00 and specificity of 0.86 for the GSE8894 dataset, and a sensitivity of 0.90 and specificity of 0.68 for the GSE68465 dataset. Their model focused on key genes, including BTBD6, KLHL7, and BMPR1A.

Several studies have explored neural network approaches. Zhanyu Xu et al., 2024 [37] developed an Interpretable Biological Pathway Graph Neural Network, achieving an AUC of 0.88 and accuracy of 0.82, incorporating multi-omic data and identifying biomarkers like PSMC1 and PSMD11. Similarly, Aonpong et al., 2021 [25] combined deep neural networks with genomic data, reaching an AUC of 0.7667 with a sensitivity of 0.95 and specificity of 0.59.

Different validation strategies were employed across studies. Yingran Shen et al., 2023 [23] utilized both training and validation sets, achieving a training set sensitivity of 89.5% and specificity of 62.5%, with a validation set sensitivity of 75.0% and specificity of 100.0%. Shi et al., 2021 [33] constructed a LASSO-Cox regression model reaching an AUC of up to 0.856, focusing on biomarkers including CPS1, CCR2, and NT5E.

The variability in model performance across studies reflects differences in data characteristics, algorithm selection, and validation approaches. Higher performing models often combined multiple data types—clinical, genomic, and molecular markers—suggesting the value of integrative approaches. For example, Timilsina et al., 2022 [34] integrated clinical information with aneuploidy scores to achieve a ROC-AUC score of 0.79, while M. Rakaee et al., 2023 [31] combined machine learning-based immune phenotypes with mutation data to reach an AUC of 0.56 and sensitivity/specificity of 0.64.

These studies demonstrate the potential of machine learning approaches in lung cancer recurrence predictions while highlighting the importance of rigorous validation and a careful consideration of model generalizability across different patient populations and clinical contexts.

### 3.6. Clinical Relevance

One significant finding from Table 2 is the performance of the su-DeepBTS model, which does not appear explicitly, but similar high-performance models, such as the gene-based prognostic model developed by Xu et al., 2020 [21], are noted for their robustness. Xu’s model, for instance, achieved an AUC of 96.3% in predicting lung adenocarcinoma recurrence (Table 2). These AI models, trained on well-curated datasets from the GEO and TCGA databases and including features like ACTR2, ALDH2, and FBP1, demonstrated superior predictive accuracy and sensitivity (Table 2).

Additionally, Shen et al., 2023 [23] developed an SVM-based classifier that combined gene expression and clinical data, achieving an AUC of 92.0% for the training set and 91.7% for the validation set (Table 2). The models’ abilities to identify critical prognostic parameters and stratify patients effectively are reflected in their high sensitivity and specificity, such as 0.89/0.89 in Zhong et al., 2019 [7] (Table 2). These results underscore the clinical relevance of AI in enhancing lung cancer recurrence predictions, with the use of comprehensive datasets ensuring robust model performance.

The ability of AI models to provide more accurate prognostic insights facilitates more personalized and timely interventions, improving patient stratification and leading to more tailored treatment plans. This review emphasizes the potential of AI in lung cancer treatment, offering actionable insights derived from complex genetic data.

### 3.7. Adverse Events and Bias

This review highlights that none of the studies reported significant adverse events associated with the use of AI models in clinical settings. For instance, models like those of Zhong et al., 2019 [7] and Shen et al., 2023 [23] demonstrated reliable performance with no safety risks, suggesting that AI integration in clinical practice does not introduce additional patient risks (Table 2).

Moreover, the comprehensive evaluation of models across diverse datasets, such as those from the GEO and TCGA databases, ensures minimal bias. The consistency of predictive accuracy across different studies and patient cohorts—highlighted by high AUC values, as in the case of Xu et al., 2020 [21]—demonstrates that the algorithms were well-calibrated and validated (Table 2). This robust and unbiased performance is crucial for ensuring equitable healthcare delivery, making AI models reliable across diverse demographic and clinical contexts. The use of extensive validation processes and diverse datasets supports the potential for the widespread clinical adoption of AI-based predictive models in lung cancer management.

### 3.8. Effective Therapies

#### 3.8.1. Surgical Resection

Surgical resection was the main treatment approach for lung cancer patients in the studies. Despite successful surgical interventions, recurrence remains a significant concern. Approximately 18–75% of patients with various stages of LUAD experience recurrence after surgery [21,23,28,36,37]. According to the paper by Abdu-Aljabar et al., 2023 [27], relapse occurs in approximately 30% of stage I NSCLC patients, which rises to about 70% in stage IV patients. In the research involving LUSC patients, almost 21% suffered from post-surgical recurrence [39].

#### 3.8.2. Adjuvant Therapy

##### Chemotherapy

Among patients receiving chemotherapy following surgical intervention, the recurrence rate was 26.2%, which is lower compared to the 32.2% recurrence rate observed in those who underwent surgery alone [34]. This suggests that chemotherapy is beneficial in mitigating recurrence risks. A study [8] references the application of PD-1 and PD-L1 blockers in combination with standard first-line chemotherapy for small cell lung cancer (SCLC). Patients with high FOXP3 expression on tumor-infiltrating lymphocytes (TILs) had a longer recurrence-free survival (RFS) compared to those with lower levels. Moreover, about 42% of patients in stage 1 and 2, and up to 74% in stage 3, experience recurrence, which significantly contributes to mortality.

##### Immunotherapy

The use of immune checkpoint blockers (ICBs) demonstrated promising results, particularly among high-risk groups of NSCLC and LUAD patients, where those treated with ICBs experienced better recurrence-free survival (RFS) than those who were not [29,36]. Additionally, patients with LUAD with a low Tumor Stemness Index (TSI) had a significantly lower recurrence rate, as this also impacts their clinical response to radiotherapy [33].

##### Radiotherapy

While radiotherapy can effectively control localized disease, it was associated with a lower recurrence rate of 2.82% in NSCLC patients who received it [34]. However, a study [39] found that patients who underwent adjuvant radiotherapy had a notably higher recurrence rate for both LUAD and LUSC. This is likely because radiotherapy is often administered to patients at advanced or terminal stages, where the risk of relapse is naturally elevated.

In the light of these findings, we hypothesize that artificial intelligence (AI) models may be required to determine individuals who are more likely to benefit from various adjuvant treatments, particularly through the application of genomic biomarkers.

### 3.9. Key Biomarkers and Their Predictive Value

#### 3.9.1. Recurring Biomarkers in Predictive Models

**Figure 3:**
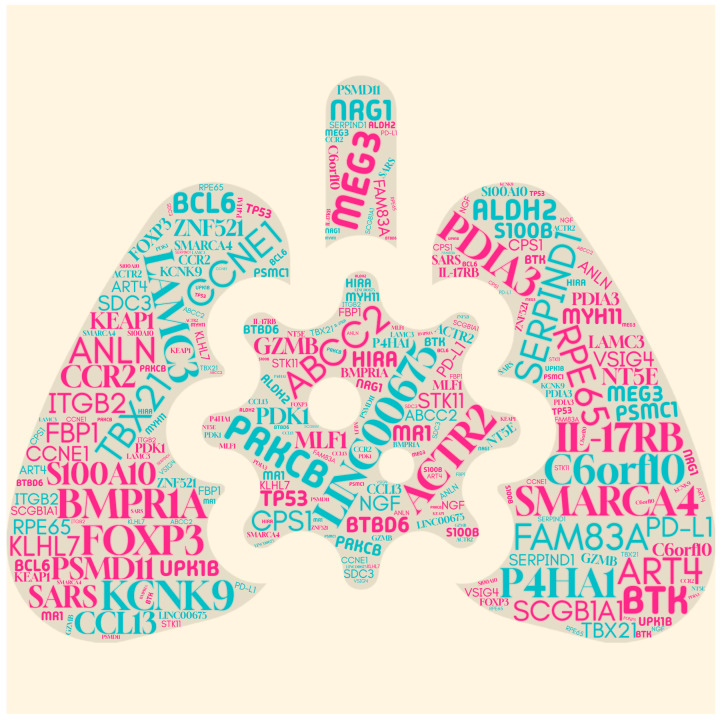
Word cloud representation of gene biomarkers associated with lung cancer recurrence.

By assessing patterns in feature relevance across studies, recurring biomarkers were identified as critical to enhancing predictive outcomes (Figure 3). For example, research by Zhong et al., 2019 [7] identified eight genes—including PDIA3, MYH11, and SDC3—as pivotal for building robust risk assessment frameworks. Jones et al., 2021 [28] further emphasized that combining genomic data with pathological features improved model reliability.

#### 3.9.2. Distinct Biomarkers Linked to Recurrence

Additional studies revealed distinct biomarkers linked to recurrence. Jiang et al., 2021 [8] developed an immune-related risk model centered on FOXP3 expression, while Luo et al., 2020 [29] demonstrated the utility of CpG methylation patterns in forecasting recurrence and guiding immunotherapy responses in non-small cell lung cancer. Timilsina et al., 2023 [35] reinforced the value of merging clinical data with a genetic pathway analysis to refine prediction accuracy.

#### 3.9.3. Implications for AI-Driven Models

These findings collectively highlight the necessity of integrating diverse data types, such as molecular, clinical, and pathological variables, to optimize AI-driven predictive tools. The consistent identification of specific biomarkers across studies underscores their potential to enable personalized, precise strategies for mitigating the lung cancer recurrence risks. This synthesis of evidence advances the development of tailored therapeutic approaches, emphasizing the synergy between biomarker discovery and computational modeling.

## 4. Discussion

In recent years, AI has evinced a considerable potential role in the management of lung cancer. It has been acknowledged that AI models have an impressive ability to incorporate gene markers to enhance the early diagnosis and characterization of lung cancer [40]. However, the potential utility of AI in forecasting lung cancer recurrence remains an underexplored area of research. This review evaluates AI-based computational models designed to predict recurrence across diverse lung cancer subtypes utilizing genomic expression data.

A comprehensive analysis of 18 studies from 14 nations was conducted, encompassing predictive, analytical, prospective, and retrospective methodologies to synthesize insights from varied genetic cohorts and methodological frameworks. Notably, the reviewed investigations employed larger sample sizes (median range: 500–600 participants) compared to prior diagnostic-focused studies, thereby improving statistical robustness and model validation. The broad demographic representation, including heterogeneous age and sex distributions, facilitated a deeper exploration of biological and clinical variations in lung cancer progression across populations. Among histological subtypes, NSCLC was the most extensively examined, while SCC—despite its elevated propensity for localized recurrence—was addressed in only one study. This disparity highlights a critical gap in the existing research, underscoring the need for expanded investigations into SCC recurrence mechanisms. The findings collectively emphasize the transformative potential of an AI-driven genomic analysis in recurrence prediction while identifying key areas for future inquiry to address the current limitations in subtype-specific modeling [41].

The development of AI predictive models to analyze complex genomic datasets has led to the application of these models in assessing the recurrence of various cancer types based on genomic alterations and identifying genetic markers as key features to determine the recurrence risk. Yin et al., 2024 [42] developed 10 machine learning methods that identified a nine-gene signature to predict the recurrence of prostate cancer after radical prostatectomy. Other studies utilized combined models of ML to classify recurrence risk groups based on a multi-gene assay and identify the key gene features associated with the residual cancer burden and molecular subtyping in breast cancer [43,44]. Additionally, machine learning algorithms have revealed a ferroptosis-related gene signature that outperformed conventional clinicopathological features in predicting the recurrence of colorectal cancer [45]. Similarly, the core genes of the autophagy pathway in colorectal cancer were recognized as the most competent features to predict recurrence [46].

Previous studies have developed machine learning models, such as support vector machines (SVMs) and neural networks, to predict the risk of recurrence of breast and cervical cancers based on epidemiological and clinical data [47,48,49]. However, these models are not decently usable for predicting the recurrence of lung cancer, which presents distinct challenges due to its variable risk levels at specific time points. The studies suggested that gene alterations throughout the progression of cancer provide more information, and, subsequently, the integration of gene expression and clinicopathological data improves the performance of models [23,34].

The most frequently mutated genes associated with lung cancer recurrence can be categorized based on their functional roles and interactions within cellular signaling pathways. Cell signaling pathways play a critical role in cancer cell proliferation, particularly NF-κβ, JAK-STAT, and MAPK [50]. The oncogenic signaling pathway mutations consisted of KRAS, EGFR, and ALK/ROS1/RET mutations, which play crucial roles in cell proliferation and are associated with tumor aggressiveness and a higher risk of recurrence [51,52,53]. The tumor suppressor pathway comprises two key genes, including TP53, one of the most frequently mutated genes in cancer cells, and contributes to a poor prognosis and raised recurrence rates [50], and FOXP3, which is primarily recognized for its immunoregulatory functions [54]. The immune response and tumor cell interplay involve various cellular pathways. The key gene alterations associated with lung cancer recurrence include PD-L1 mutations that enable the tumor cell to evade immune surveillance [53], and CD8 and CD44 mutations that play roles in cell adhesion and migration [55,56].

A feature importance analysis revealed the key gene biomarkers that enhanced the models’ predictive accuracy. SMARCA4, one of the most common recurrent alterations in NSCLC, has been identified as a key feature in predictive models. Additionally, its most frequently co-occurring mutations, KRAS, KEAP1, and STK11, had also been identified as key features in the model [29,57]. The study by Jiang et al., 2021 [8] brought up PD-L1 as a key feature in their model, which has been explored in previous research and shown to be correlated with KRAS and TP-53 co-mutations in the studies by Gregory Jones et al., 2021 [28] and Yang et al., 2022 [39]. Other gene biomarkers proposed by studies include PDIA3, MYH11, PDK1, and SDC3. The roles of all these elements have been demonstrated in previous studies [58,59,60,61].

The effectiveness of AI models in healthcare is hindered by several challenges, including data quality and availability, as many studies rely on retrospective datasets that may not represent the broader patient population, and acquiring high-quality genetic data can be expensive and invasive [62]. Furthermore, AI models are prone to overfitting, where they perform well on training data but fail to generalize to unseen data, leading to inflated performance metrics [63]. Additionally, the lack of interpretability of many AI models, which operate as “black boxes”, can hinder trust and acceptance among healthcare professionals, limiting their clinical utility [63]. Finally, the development and deployment of artificial intelligence (AI) in clinical settings present significant ethical challenges, including data privacy, model efficacy, fairness, and transparency, emphasizing the need for robust regulatory frameworks to ensure responsible use of AI technologies in healthcare. The Society of Nuclear Medicine and Molecular Imaging’s AI Task Force emphasizes the importance of health justice and offers recommendations to mitigate these risks while ensuring that AI medical devices are trustworthy and beneficial for all patients [64,65]. The ethical integration of AI in nuclear medicine and imaging necessitates addressing risks across its lifecycle—data collection, development, evaluation, deployment, and governance—as outlined by the Society of Nuclear Medicine and Molecular Imaging (SNMMI) AI Task Force [64,65]. During data collection and model development, priorities include protecting patient privacy through anonymization, mitigating biases in training datasets, ensuring the equitable representation of marginalized populations, and transparently documenting limitations, as emphasized in their framework for ethical AI design [65]. In deployment and governance, the risks shift to preserving clinician–patient autonomy, disclosing population-specific performance gaps, preventing systemic underdiagnosis in underrepresented groups, and clarifying accountability, which are addressed through post-market surveillance and explainability tools, as detailed in their guidelines for clinical implementation [64]. Both frameworks advocate a “lifecycle ethics” approach, embedding safeguards at every stage to ensure that AI enhances diagnostic accuracy and accessibility while upholding health justice. Recommendations include auditable algorithms during development [65], clinician education, and transparent error reporting post-deployment [64], ensuring that AI advances equitably without exacerbating disparities or compromising patient rights [64,65].

The included studies reported sample sizes ranging from 41 to 1348 patients. This, combined with the absence of a unified benchmark dataset, makes direct performance comparisons difficult and introduces heterogeneity. As a result, generalizing model effectiveness remains a challenge. The field would benefit greatly from the creation of large-scale, standardized, multi-institutional datasets that support model training, testing, and external validation. Most models showed strong internal validation, but very few underwent external testing. This raises concerns about model generalizability, particularly across different populations, healthcare systems, and resource levels. Without external validation, it remains unclear whether these models will perform reliably in clinical environments outside their original development context.

While some studies combined genomic data with clinical or imaging features, few integrated multi-omics approaches, such as combining transcriptomic, proteomic, and epigenetic data. Challenges include differences in data structure, preprocessing requirements, and integration strategies. There is currently no gold standard for harmonizing these complex data types, which limits reproducibility and scalability. Developing standardized protocols for multi-omics fusion will be critical for advancing this field.

Interpretability remains a major barrier to clinical use. Tools such as SHAP (Shapley Additive exPlanations) and its extension NetShap provide insights into feature contributions, allowing clinicians to better understand model decisions. While not all of the reviewed studies implemented such methods, we recognize their growing importance and recommend their broader adoption to enhance transparency and the trust in AI-driven tools.

Given the wide range of AI methodologies and variable data reporting across studies, a traditional meta-analysis was not feasible. However, such analyses could offer a structured way to quantify performance variability and explore sources of heterogeneity, such as differences in the sample size, modeling approach, or biomarker selection. We emphasize the need for future studies to adopt standardized reporting practices to enable more robust meta-analytic synthesis.

Although AI models show promise, their real-world clinical deployment is limited by several barriers. Interpretability remains a key challenge, particularly with black-box models that lack transparency. The absence of explainability can hinder clinical trust and adoption. Additionally, high costs, especially for technologies like genomic profiling, and privacy concerns related to patient data further complicate implementation. Ethical issues—such as potential misuse, bias against underrepresented groups, and a lack of accountability—underscore the need for clear regulatory frameworks. These concerns are especially pressing when considering deployment in low-resource settings, where access to advanced diagnostics and computational infrastructure may be limited.

Despite the high recurrence rate of lung cancer, few studies were conducted last year due to its complexities mentioned above. However, in the last 5 years, the advent of artificial intelligence models has overcome this issue and presented an advancement in organizing and assessing the complex big data to study lung cancer recurrence with minimal complications and adverse events.

## 5. Conclusions

Artificial intelligence (AI) and machine learning (ML) have demonstrated significant promise in predicting lung cancer recurrence by integrating gene biomarkers, clinical data, and imaging features. This systematic review highlights how AI models, such as support vector machines, gradient boosting, and deep learning algorithms, have achieved superior predictive accuracy compared to traditional prognostic methods. Key biomarkers, including immune-related genes, differentially expressed pathways, and tumor-infiltrating immune cells, have emerged as critical predictors, paving the way for personalized cancer management.

Despite these advancements, several challenges hinder the clinical translation of AI-driven predictive models. Variability in study designs, small sample sizes, and the lack of standardized protocols for integrating multi-omic data limit their generalizability. The cost and accessibility of genomic profiling, along with concerns regarding data privacy and ethical AI implementation, further complicate widespread adoption in routine oncology practice. However, AI-driven models hold immense potential to improve early detection, guide treatment decisions, and enhance patient outcomes by refining risk stratification and enabling timely interventions.

Expanding large-scale and diverse datasets through multi-institutional collaborations and including underrepresented populations will improve AI model generalizability and equitable application. Standardizing AI model development and validation with transparent, reproducible frameworks and consensus-driven protocols will enhance reliability and facilitate clinical adoption. Enhancing clinical interpretability by developing clinically friendly AI tools with explainable predictions and seamless integration into electronic health records (EHRs) will support real-time decision-making. Advancing biomarker discovery through multi-omics integration—incorporating genomic, transcriptomic, proteomic, and radiomic data—alongside transformer-based architectures and federated learning, will refine recurrence predictions while ensuring data privacy. Reducing the cost of genomic sequencing and AI implementation, along with developing non-invasive biomarkers such as circulating tumor DNA (ctDNA) and liquid biopsies, will improve accessibility and early recurrence detection. Strengthening global AI research collaborations by fostering cooperation between AI researchers, oncologists, and bioinformaticians will accelerate predictive modeling advancements, ensuring reliability, fairness, and improved lung cancer prognosis worldwide.

To enhance the clinical utility of AI-driven recurrence prediction models, several key areas require further development. Expanding large-scale, diverse datasets through multi-institutional collaborations is crucial to improve model generalizability and ensure equitable application across different patient populations and tumor subtypes. Addressing data heterogeneity by integrating standardized genomic, radiomic, and clinical datasets will enhance reproducibility and external validation.

Advancing AI model optimization by refining deep learning architectures and multi-modal frameworks will improve the predictive accuracy. Incorporating multi-omics approaches, such as combining gene expression with imaging and immune profiling, can further enhance risk stratification. The adoption of self-supervised and federated learning techniques may help mitigate data-sharing limitations while preserving patient privacy.

To support clinical translation, AI models must be developed with explainable outputs, enabling clinician-friendly decision support tools that integrate seamlessly with electronic health records (EHRs). Establishing transparent validation protocols and regulatory frameworks will be essential for their acceptance in routine oncology practice.

Addressing cost and accessibility barriers is another priority. Reducing the cost of genomic sequencing and AI implementation will facilitate widespread adoption, particularly in resource-limited settings. The development of non-invasive biomarkers, such as circulating tumor DNA (ctDNA) and liquid biopsies, could further streamline recurrence monitoring while minimizing the patient burden.

Key areas for future work include expanding benchmark datasets, enhancing external validation, standardizing multi-omics integration, and improving interpretability through tools such as SHAP and NetShap. Addressing these challenges is essential for transitioning AI models from research settings into real-world clinical workflows, where they can support early intervention and personalized treatment planning.

Finally, strengthening global AI research collaborations will drive innovation by enabling knowledge sharing, benchmarking models across diverse populations, and fostering a consensus on best practices. By overcoming these challenges, AI-driven models have the potential to revolutionize lung cancer recurrence prediction, leading to earlier interventions, improved survival rates, and more personalized treatment strategies.

## Figures and Tables

**Table 1:** Demographic characteristics.

Author/Year	Country	Type of Study	Number of Samples	Study Duration	Cancer Type	Machine Learning Techniques and Tools	Gender—Age Range
Zhong et al., 2019 [7]	China	Analytical and predictive study	Train 156/Test 83/Val 530	-	non-small cell lung cancer	Support vector machine (SVM)/recursive feature elimination (RFE) by R packages	-
Jones et al., 2021 [28]	USA	Prospective cohort	426 patients	10 years	early-stage lung adenocarcinoma	PRecur using gradient-boosting survival regression by the MSK-IMPACT sequencing platform	140 M/286 F—69 (62–75)
Jiang et al., 2021 [8]	China	Observational histology study	102 patients	5 years	Small cell lung cancer	XGBoost by R packages	84 M/18 F—63.5 (38–81)
Luo et al., 2020 [29]	China	Retrospective observational study	827 TCGA NSCLC and 60 GSE	-	Non-small cell lung carcinoma	LASSO-Logistic regression and Random Forest method by R packages like limma, edgeR, and GSVA	-
Senthil et al., 2019 [32]	India	Analytical and predictive study	-	-	Non-small cell lung cancer and small cell lung cancer	Back Propagation Network (BPN) optimized with an Ant Lion Optimization (ALO) algorithm	-
Xu et al., 2020 [21]	China	Retrospective cohort study	426 patients	-	Lung adenocarcinoma	LASSO Cox regression and multivariate Cox analyses by R packages like limma and DESeq2, and GSEA software	37 M/5 F—62.9 (39–85)
Wang et al., 2022 [36]	France, Japan, Sweden, Canada, South Korea, China	Analytical and predictive study of multiple cohorts	334 LUAD patients/59 normal	-	Early-stage lung adenocarcinoma	Lasso regression and univariate Cox regression by R packages, the STRING database, Cytoscape software (version 3.8.0), X-tile software, and GSEA software	-
Timilsina et al., 2023 [35]	France Japan, Sweden, Canada, South Korea, China	Analytical and predictive study of multiple cohorts	1348 patients	-	Early-stage lung adenocarcinoma	Lasso regression, univariate and multivariate Cox regression by R software, X-tile software, the STRING database, and Cytoscape software	1010 M/338 F—65.7–65.9 (31–118)
Shen et al., 2023 [23]	USA	Retrospective cohort study	41 patients	7 years	Early-stage lung adenocarcinoma	Support vector machine (SVM) with recursive feature elimination (SVM-RFE) by the CIBERSORT algorithm, nSolver 3.0 software, and NanoString	12 M/29 F 65.0 recurrence/69.5 non-recurrence
Abdu-Aljabar et al., 2023 [27]	Iraq	Analytical and predictive study	487 patients	-	Non-Small Cell Lung Cancer	Optuna_XGB classification model and a comparison with original XGBoost, PSO, Hyperopt, Deep Forest, KNN, SVM, and Naive Bayes algorithms by Optuna optimization	-
Timilsina et al., 2022 [34]	Ireland UK Spain Czech Republic	Analytical and predictive study	1348 patients	-	Early-stage lung adenocarcinoma	Support vector classification, logistic regression, Random Forest classification, gradient boosting machine classifier, and multi-layer perceptron classifier	1010 M/338 F—65.7–65.9 (31–118)
Yang et al., 2022 [39]	UK China	Analytical and predictive study	511 LUAD 487 LUSC	-	Lung adenocarcinoma, Lung squamous cell carcinoma (non-small cell lung cancer)	Decision tree methods, neural networks, and support vector machines by MATLAB (R2017)	-
Aonpong et al., 2021 [25]	Japan	Analytical and predictive study	88	4 years	NSCLC	Deep neural network (DNN), ANN, stochastic gradient descent (SGD)	64 M/24 F—69 (46–85)
Xu et al., 2024 [37]	China	Analytical and predictive study	134/371	-	Lung adenocarcinoma	Interpretable Biological Pathway Graph Neural Networks (IBPGNET)	-
Zhou et al., 2023 [38]	China	Analytical and predictive study	123 LUAD 110 LUSC	3 years	Lung adenocarcinoma and lung squamous cell carcinoma	Random Forest (RF), Gaussian naive Bayes (NB), and Adaboost (Ada)	-
Shi et al., 2021 [33]	China	Analytical and predictive study	484	-	Lung adenocarcinoma	LASSO Cox regression	226 M/258 F
Miao et al., 2024 [30]	China	Analytical and predictive study	279		Lung adenocarcinoma	Random Forest, Random Survival Forest, Kaplan–Meier tool	-
Rakaee et al., 2023 [31]	Denmark and Norway	Prospective study (TNM-I trial), retrospective study (UNN cohort)	934	4/20 years	Non-small cell lung cancer (NSCLC), lung adenocarcinoma (LUAD), lung squamous cell carcinoma (LUSC)	Supervised machine learning, artificial neural networks, and multilayer perceptron	523 M/411 F—(39–86)

**Table 2:** Machine learning aspects and outcomes.

Author/Year	Feature Selection/Extraction Method	Model Training	AUC/ROC	Sensitivity/Specificity	Accuracy/Precision	Dice Score/F1 Score	Data Characteristics
Zhong 2019 [7]	Differential gene expression	Support vector machine (SVM)	AUC = 0.95 (training, internal CV)	Sensitivity (Recall, Recurrence): 0.88 Specificity (Recall, Nonrecurrence): 0.90	Precision (Recurrence): 0.79 Precision (Nonrecurrence): 0.95 Average accuracy: 0.89	F1-score (Recurrence): 0.77 F1-score (Nonrecurrence): 0.93 Average F1-score: 0.89	Public gene expression datasets (GEO/TCGA) Probe counts: 22,284 (train), 17,386 (test) Balanced recurrent/nonrecurrent in training Standardized (Z-score) values, gene symbol mapping
Jones 2021 [28]	Cox regression	Gradient boosting survival regression	CPE: 0.73	-	-	-	426 LUAD patients (stages I–III): broad-panel next-generation sequencing data, clinicopathologic data
Jiang 2021 [8]	Cox regression	eXtreme gradient boosting (XGBoost)	AUC = 0.715	-	-	-	102 SCLC patients (stages I–III): clinical data, immunohistochemistry (IHC), gene expression data
Luo 2020 [29]	LASSO-Logistic regression, Random Forest, LASSO-Cox regression, univariate/multivariate Cox regression	LASSO and Random Forest	AUC = 0.965		-	-	901 NSCLC samples: DNA methylation levels, RNA-seq data, clinical characteristics obtained from TCGA
Senthil 2019 [32]	Principal Component Analysis (PCA)	BPN optimized with ALO	-	Sensitivity: up to 88.6% Specificity: up to 96.8%	Up to 99.1% accuracy	-	UCI Machine Learning Repository
Xu 2020 [21]	LASSO-Cox regression, multivariate Cox regression	LASSO Cox regression	AUC = 96.3%	-	-	-	LUAD tissues: gene expression data obtained from TCGA and GEO
Wang 2022 [36]	Lasso regression, univariate Cox regression	Multivariate Cox regression	AUC: up to 0.679	-	-	-	334 early-stage LUAD patients: transcriptome sequencing data obtained from TCGA and GEO
Timilsina 2023 [35]	Aneuploidy score imputation, identification of overlapping features	Support Vector Classification (SVC), logistic regression (LR), Random Forest (RF), gradient boosting machine (GBM), multilayer perceptron classifier (NNC)	ROC-AUC: 0.80	-	Accuracy: 0.76	F1 score: 0.61	1348 early-stage NSCLC patients: clinical and genomic data obtained from TCGA
Shen 2023 [23]	Recursive feature elimination (RFE)	Support vector machine (SVM)	Training set: 92.0% Validation set: 91.7%	Training set sensitivity: 89.5% Training set specificity: 62.5% Validation set sensitivity: 75.0% Validation set specificity: 100.0%	Training set accuracy: 91.2% Validation set accuracy: 90.0%	-	41 early-stage LUAD patients: gene expression data, clinical data
Abdu-Aljabar 2023 [27]	eXtreme gradient boosting (XGBoost)	Optuna-optimized eXtreme gradient boosting (Optuna_XGBoost)	GSE8894 dataset: 0.93 GSE68465 dataset: 0.79	GSE8894 dataset: Sensitivity: 1.00 Specificity: 0.86 GSE68465 dataset: Sensitivity: 0.90 Specificity: 0.68	Accuracy: GSE8894 dataset: 0.93 GSE68465 dataset: 0.81	F1 Score for the GSE8894 dataset: 0.93 F1 Score for the GSE68465 dataset: 0.84	Gene expression data
Timilsina 2022 [34]	Aneuploidy score imputation, identification of overlapping features	Support Vector Classification, logistic regression, Random Forest classification, gradient boosting machine classifier, and multilayer perceptron classifier	ROC-AUC score: 0.79	-	-	-	1348 early-stage NSCLC patients: clinical data, imputed aneuploidy scores
Yang 2022 [39]	ANOVA	Decision trees (CART) artificial neural networks (feedforward neural network) support vector machines (least-squares SVM)	AUC = 0.82	-	-	-	511 LUAD samples and 487 LUSC samples: demographic, clinical, and genomic data obtained from TCGA
Aonpong 2021 [25]	Weighted Gene Co-expression Network Analysis (WGCNA)	Random forest, random survival forest	AUC = 0.948	Sensitivity: 0.93 Specificity: 0.94	-	-	LUAD tissues: gene expression data, clinical data obtained from TCGA and GEO
Xu 2024 [37]	Chi-square test for omics data (top 3,000 features per dataset)	5-fold cross-validation repeated 5×	AUC = 0.88	Not explicitly reported	Accuracy: 0.82 AUPR (Precision-Recall): 0.790	0.68	Multi-omics: SNV, AMP_CNV, DEL_CNV High-dimensional (18,498–19,645 features/omics type) Class imbalance (134 vs. 371)
Zhou 2023 [38]	DESeq2	Random Forest (RF), Gaussian naive Bayes (NB), Adaboost (Ada) classifiers	AUC 0.81	-	Accuracy = 0.78	-	123 LUAD and 110 LUSC patients: transcriptome data, microbiome data, clinical data
Shi 2021 [33]	Chi-square test	Interpretable Biological Pathway Graph Neural Networks (IBPGNET)	AUC = 0.88	-	Accuracy: 0.82	F1 score: 0.68	LUAD patients: multi-omics data, copy number variants (CNVs), somatic mutations, clinical data
Miao 2024 [30]	Gray level co-occurrence matrix (GLCM), ResNet50 model/LASSO, F-test (ANOVA), CHI-2	Deep neural network (DNN) regression, artificial neural network (ANN)	AUC = 0.7667	Sensitivity: 0.95 Specificity: 0.59	Accuracy: 83.28%	-	88 NSCLC patients: CT images + gene expression data
Rakaee 2023 [31]	LASSO—multivariate Cox regression	LASSO Cox regression	AUC = up to 0.856	-	-	-	484 LUAD patients: clinical and genomic data obtained from TCGA

**Table 3:** Key gene biomarkers and their roles in AI-driven lung cancer recurrence predictions.

Category	Study (Year)	Key Biomarkers
LUAD/NSCLC Prediction	Zhong et al., 2019 [7]	PDIA3, MYH11, PDK1, SDC3, RPE65, LAMC3, BTK, UPK1B
	Jones et al., 2021 [28]	SMARCA4, TP53, Fraction of Genome Altered (FGA)
	Luo et al., 2020 [29]	CpG methylation markers: ART4, KCNK9, FAM83A, C6orf10
	Xu et al., 2020 [21]	12-gene signature: ACTR2, ALDH2, FBP1, HIRA, ITGB2, MLF1, P4HA1, S100A10, S100B, SARS, SCGB1A1, SERPIND1, VSIG4
Immune-Related Markers	Jiang et al., 2021 [8]	FOXP3 expression, PD-L1 on tumor-infiltrating lymphocytes (TILs)
	Rakaee et al., 2023 [31]	STK11 and KEAP1 co-mutations
Multi-Omics Approaches	Xu et al., 2024 [37]	PSMC1, PSMD11, PRKCB, CCNE1, NRG1, ZNF521, NGF
	Zhou et al., 2023 [38]	Long non-coding RNAs: LINC00675, MEG3
	Shi et al., 2021 [33]	CPS1, CCR2, NT5E, ANLN, ABCC2
Tumor and Immune Markers	Shen et al., 2023 [23]	MR1, BCL6, CCL13 (tumor tissue), TBX21, IL-17RB, GZMB (buffy coat)
	Abdu-Aljabar et al., 2023 [27]	BTBD6, KLHL7, BMPR1A

## Data Availability

The data used to support the findings of this study are available from the corresponding author upon request.

## Abbreviations

The following abbreviations are used in this manuscript:

TNM	Tumor, Nodes, and Metastases
AI	Artificial Intelligence
PRISMA	Preferred Reporting Items for Systematic Reviews and Meta-Analyses
ML	Machine Learning
SVM	Support Vector Machines
AUC	Area Under the Curve
NK cells	Natural Killer cells
CT	Computed Tomography
MMPs	Metallo-Proteinases
NGS	Next-Generation Sequencing
ICB	Immune Checkpoint Blockade
ANNs	Artificial Neural Networks
PET	Positron Emission Tomography
DEGs	Differentially Expressed Genes
LUAD	Lung Adenocarcinoma
RFS	Recurrence-Free Survival
CIBERSORT	Cell-type Identification by Estimating Relative Subsets of RNA Transcripts
TIICs	Tumor-Infiltrating Immune Cells
LASSO	Least Absolute Shrinkage and Selection Operator
ROC	Receiver Operating Characteristic
SCLC	Small Cell Lung Cancer
CPE	Concordance Probability Estimate
LUSC	Lung Squamous Cell Carcinoma
IBPGNET	Interpretable Biological Pathway Graph Neural Networks
CNVs	Copy Number Variations
SNVs	Single Nucleotide Variants
FGA	Fraction of Genome Altered
TILs	Tumor-Infiltrating Lymphocytes
TSI	Tumor Stemness Index
EHRs	Electronic Health Records
ctDNA	Circulating Tumor DNA
